#  Hospital-Based Cancer Incidence in Nepal from 2010 to 2013 

**DOI:** 10.3126/nje.v7i1.17759

**Published:** 2017-03-31

**Authors:** Krishna Kanta Poudel, Zhibi Huang, Prakash Raj Neupane, Roberta Steel, Janaki Kharel Poudel

**Affiliations:** 1 Researcher, Bhaktapur Cancer Hospital, Nepal.; 2 Professor, Department of Epidemiology and Biostatistics, School of Public Health Guangxi Medical University, China; 3 Chairman, B P Koirala Memorial Cancer Hospital, Bharatpur Chitwan, Nepal; 4 Quality Consultant, Cavendish Square Wellington, New Zealand; 5 Researcher, Community Health Campaign, Chitwan ,Nepal

**Keywords:** Cancer, male and female, site, incidence, Nepal.

## Abstract

**Background:**

Cancer is one of the leading causes of death throughout the world. Analyzing the incidence of cancer by site, sex and age is essential to detect the burden of cancer. Throughout the twelve hospital based cancer registries of Nepal, a total of 29,802 cancer cases with known age, were registered from January 1st 2010 to 2013 December 31st. The purpose of this retrospective study is to present the incidence of all cancer sites in both males and females for this period.

**Materials and Methods:**

This paper reviews data from all the hospital based cancer registries over a four-year period. This retrospective study has illustrated the number of cases, frequencies and crude incidence of all cancers by sex and site. For statistical analysis, SPSS (version 23.0) and Microsoft Excel 2010 were used.

**Results:**

Over the four-year period from January 1st 2010 to 2013 December 31st the major cancer in males was identified as follows: lung cancer (17.5%) followed by stomach cancer (7.6 %) and larynx cancer (5.4%). Among females, for the same four-year period, the three common cancers were identified as cervix (18.9 %) followed by breast (15.6 %) and lung (10.2%).

**Conclusion:**

This retrospective study concluded that cancer is being increased by calendar years both in males and females however, the incidence of cancer is higher in females compared to males. .

## Introduction

Population based cancer registry is currently unavailable in Nepal although there are twelve hospital based cancer registries (B P Koirala Memorial Cancer hospital, Bhaktapur Cancer Hospital, Bir hospital, TU Teaching Hospital, Kanti Children Hospital, BP Koirala Institute of Health Science and Manipal Teaching Hospital, Shree Birendra Hospital, Civil Service Hospital, Patan Hospital, Paropakar Maternity and Women Hospital and Nepalgunj Teaching Hospital) operating throughout this country [ 1 ]. The first hospital-based cancer registry was established in 2003 with the aid of the World Health Organization. Documents were previously issued which highlighted the problem of cancer cases in Nepal [ 2, 3, 4, 5 ], however, owing to the lack of community based cancer registries, we have taken the population (denominator) from the census and cases from the hospital. This study primarily relies on the analysis of data from all the cancer registries in Nepal between 2010-2013 inclusive. In so doing, it has demonstrated the reality that hospital based study remains incomplete due to a lack of data because many people either remain incapable to visit hospitals or they are unaware of cancer. For any meaningful progress especially the reduction of cancers generally, it is imperative to secure the establishment of population based cancer registry in Nepal. The output of this study suggests there is an urgent necessity for cancer epidemiologists, statisticians as well as concerned stakeholders to invest in a preventative program to control and thereby prevent the increasing trend of cancers in Nepal.

## Methodology

Data of cancer cases was collected from all the hospital based cancer registries of Nepal between 1st January 2010 to 31st December 2013. All double/multiple entry cases were excluded by cross checking each patients name, sex, address and hospital registered number. We analyzed 13789 male cases and 16013 female cases which have known age. The collected data was entered in Excel Sheet with respect to age, sex, years and site. The population growth rate from 2001 to 2011 published in population monograph of Nepal volume 1 and the Census population of 2011 was used to estimate the 2010, 2012 and 2013 population of Nepal [ 6 ]. Cancer cases were categorized as per International Classification of Disease for Oncology (ICD-10) published by the International Agency for Research on Cancer/ World Health Organization (IARC/WHO). The crude incidence rate of cancer (per 100,000) in males and females of all sites are calculated. For statistical analysis, SPSS (version 23.0) and Microsoft Excel 2010 were used.

## Results

Lung cancer has the highest incidence (4.6) in males followed by stomach cancer (2.0) and larynx cancer (1.4) per 100,000 population from 2010 to 2013. Similarly, cervical cancer has the highest incidence (5.5) per 100,000 population in females followed by breast cancer (4.6) and lung cancer (3.0) over the same four-year period. The crude incidence of female cancer cases (29.2) per 100,000 population is higher than the crude incidence of male cancer cases (26.5) (Table 2).

**Figure 1:**
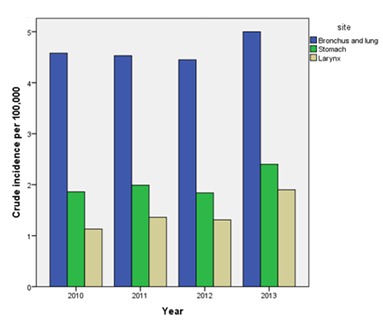
Figure 1: Crude incidence of three major cancers in males from 2010 to 2013.

**Figure 2:**
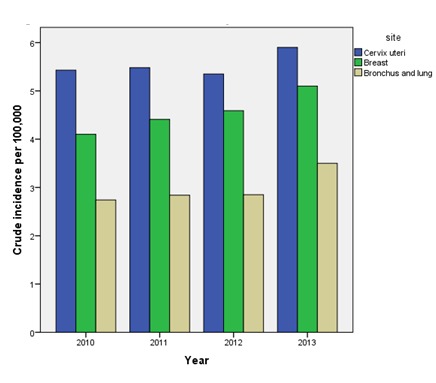
Figure 2: Crude incidence of three major cancers in females from 2010 to 2013

Cancer both in males and females is increasing by calendar years(Table 1) The incidence of three major cancers in males and females over the four-year period has been demonstrated in figure 1 and figure 2 respectively

## Discussion:

This study was undertaken in Nepal between 2010-2013 and presents the detail of cancer incidence by gender, sites and calendar years. Trends show that the cancer incidence is increasing every year especially for females. This is possibly due to increasing activities of screening programs in different areas of the country [ 7 ] and the support from the International Agency for Research on Cancer (IARC) for early detection and prevention of cervix and breast cancer [ 8 ]. Due to an increasing number of cancer diagnostic centers in Nepal, many people now also come forward to report the cancer cases which could be another possible cause for the growth of new cases of cancer in Nepal [ 9 ].

As a result of our studies we have found that bronchus and lung cancer is the most common cancer in males and the third common cancer in females. The crude incidence rate of lung cancer is 4.6 per 100,000 populations in Nepal while it was 4.45 in 2012 [ 5 ]. The following mitigating factors may have an effect on this trend: lower education, unmarried individuals and Rai/Limbu/Magar ethnicity [ 10 ], household air pollution and tobacco consumption [ 11, 12 ], and not enough medical health education [ 13 ]. For young people in western Nepal; smoking was a serious issue [ 14 ] and the risk factor leading to lung cancer [ 15 ]. The crude incidence rate of lung cancer is 3.0 per 100,000 populations in Nepal while it was 2.85 in previous years [ 5 ]. 

 Cervical uteri cancer is the most common type of cancer in females. The crude incidence rate of cervical uteri is 5.5 per 100,000 population in Nepal while it was 5.35 in 2012 [ 5 ]. Women do not have enough information regarding the human papillomavirus (HPV), cervical cancer and HPV vaccine [ 16 ], or concept of pap smear test [ 17 ]. Using the HPV16/18 vaccines, almost 80% of cervical cancer in Nepal could be prevented [ 18 ]. 

 Breast cancer is the second most common cancer in females with more than a quarter occurring in young females. The more aggressive biological features of tumors were found in this at-risk group therefore we conclude that breast cancer prevention programs are essential to reduce this trend [ 19 ]. The crude incidence rate of breast cancer is (4.6) per 100,000 populations in Nepal while it was 4.59 in 2012 [ 5 ]. 

 Ovarian cancer is the fourth most common cancer in females over this four-year period. The incidence of ovarian cancer is (1.9) per 100,000 populations while it was (1.70) in 2012 ( [ 5 ]. 

 Stomach cancer is the second most common cancer in males while it is the fifth common cancer in females for the same period. The crude incidence rate of stomach cancer in males is (2.0) per 100,000 populations in Nepal, while it was 1.84 in 2012 [ 5 ]. 

 Analyzing the data from B P Koirala Memorial Cancer Hospital, out of 7212 (7.3%) presented as stomach cancer [ 3 ]. Similarly, another study in 2005 reported out of 4397 cases (7.5%) represented male stomach cancer rates while the corresponding percentage of females was (4.1%) [ 2 ]. In female, the incidence of stomach cancer was 1.2 per 100,000 population over the four year period while it was 1.06 in 2012 [ 5 ]. Stomach cancer incidence rated men almost two times higher as compared to women although there is a worldwide variation in trend [ 15 ]. 

 Larynx cancer was the third most common cancer in males over the four-year period 2010 - 2013. The crude incidence rate of larynx cancer is (1.4) per 100,000 populations, while it was 1.0 in 2012 [ 5 ]. Out of 7212 cases in 2012 (5.2%) were the larynx cases [ 3 ]. Similarly, another study in 2005 reported out of 4397 cases, 3.7% presented as larynx cancer in males while the corresponding percentage in females was 1.3% cases [ 2 ]. 

Ovarian cancer (1.9 per 100,000 populations) is the fourth most common cancer in females while gall bladder (1.1 per 100,000 population) represents the fifth most common cancer in Nepal throughout the four-year period 2010 - 2013. Bladder cancer (1.3 per 100,000 population) is the fourth most common cancer in males while cancer of other and unspecified parts of mouth is the fifth most common cancer in Nepal for the same period. 

 In conclusion cancer incidence in Nepal is growing at a higher rate for both males and females. The most common cancers in males are bronchus and lung, stomach and larynx while in females cervical uteri, breast, bronchus and lung are the most common cancers.

### What is already known on this topic:

A couple of studies had been conducted to demonstrate the incidence of cancer in Nepal which had only focused the single calendar year.

### What this study adds:

This study is different from the previous studies in Nepal because it has clearly presented the cancer incidence of all sites over the recent four years. Furthermore, this study also performed the incidence of three major cancers in males and females from 2010 to 2013.

## Figures and Tables

**Table 1:** Table 1. Summary of cancer cases and incidence per 1, 00,000 people in Nepal from 2010 to 2013.

**Years**	**Total male cases**	**Total Female cases**	**Male crude incidence per 100,000**	**Female crude incidence per 100,000**
**2010**	3192	3581	24.8	27.8
**2011**	3295	3793	26.6	27.5
**2012**	3291	3921	25.2	28
**2013**	4011	4718	30.4	33.3

**Table 2:** Table 2. Distribution of cancer site in males and females in Nepal from 2010 to 2013.

	Male			Female		
			(Per 100,000)			(Per 100,000)	Site
ICD-10	cases	Fre (%)	Crude rate	cases	Fre (%)	Crude rate	
C00	70	0,5	0,1	24	0,1	0,0	Lip
C01	26	0,2	0,1	6	0,0	0,0	Base of Tongue
C02	348	2,5	0,7	184	1,1	0,3	Other and unspecified parts of tongue
C03	75	0,5	0,1	29	0,2	0,1	Gum
C04	60	0,4	0,1	16	0,1	0,0	Floor of mouth
C05	57	0,4	0,1	38	0,2	0,1	Palate
C06	472	3,4	0,9	161	1,0	0,3	Other and unspecified parts of mouth
C07	92	0,7	0,2	53	0,3	0,1	Parotid gland
C08	60	0,4	0,1	48	0,3	0,1	Other and unspecified major salivary gland
C09	93	0,7	0,2	36	0,2	0,1	Tonsil
C10	90	0,7	0,2	34	0,2	0,1	Oropharynx
C11	173	1,3	0,3	93	0,6	0,2	Nasopharynx
C12	259	1,9	0,5	39	0,2	0,1	Pyriform sinus
C13	44	0,3	0,1	11	0,1	0,0	Hypopharynx
C14	54	0,4	0,1	16	0,1	0,0	Other and ill-defined sites in lip oral cavity and mouth
C15	362	2,6	0,7	233	1,5	0,4	Esophagus
C16	1047	7,6	2	640	4,0	1,2	Stomach
C17	53	0,4	0,1	44	0,3	0,1	Small intestine
C18	347	2,5	0,7	277	1,7	0,5	Colon
C19	21	0,2	0	13	0,1	0,0	Rectosegmoidjucnction
C20	396	2,9	0,8	338	2,1	0,6	Rectum
C21	40	0,3	0,1	27	0,2	0,0	Anus and anal canal
C22	367	2,7	0,7	283	1,8	0,5	Liver
C23	292	2,1	0,6	577	3,6	1,1	Gall bladder
C24	66	0,5	0,1	73	0,5	0,1	Other and unspecified billary tract
C25	158	1,1	0,3	129	0,8	0,2	Pancreas
C26	15	0,1	0	6	0,0	0,0	Ill defined digestive organs
C30	85	0,6	0,2	75	0,5	0,1	Nasal cavity and middle ear
C31	74	0,5	0,1	73	0,5	0,1	Accessory sinus
C32	741	5,4	1,4	243	1,5	0,4	Larynx
C33	5	0	0	1	0,0	0,0	Trachea
C34	2414	17,5	4,6	1634	10,2	3,0	Bronchus and lung
C37	29	0,2	0,1	12	0,1	0,0	Thymus
C38	34	0,2	0,1	20	0,1	0,0	Heart, mediastinum and pleura
C39	2	0	0	2	0,0	0,0	Ill defined respiratory system
C40	167	1,2	0,3	107	0,7	0,2	Bones joint and articular cartilage
C41	173	1,3	0,3	133	0,8	0,2	Other and unspecified bones joints and articular cartilage
C42	0	0	0	2	0,0	0,0	Spleen
C43	94	0,7	0,2	71	0,4	0,1	Skin melanoma
C44	113	0,8	0,2	107	0,7	0,2	Skin other
C47	7	0,1	0	13	0,1	0,0	Perepheral nerves and autonomic nervous system
C48	36	0,3	0,1	21	0,1	0,0	Peritoneum and retroperitoneum
C49	241	1,7	0,5	188	1,2	0,3	Connective subcutaneous and other soft tissue
C50	71	0,5	0,1	2505	15,6	4,6	Breast
C51	0	0	0	107	0,7	0,2	Vulva
C52	0	0	0	98	0,6	0,2	Vagina
C53	0	0	0	3033	18,9	5,5	Cervix uteri
C54	0	0	0	214	1,3	0,4	Endometrium
C55	0	0	0	46	0,3	0,1	Uterus
C56	0	0	0	1035	6,5	1,9	Ovary
C57	0	0	0	118	0,7	0,2	Other and unspecified female genital organs
C58	0	0	0	1	0,0	0,0	Placenta
C60	230	1,7	0,4	0	0,0	0,0	Penis
C61	248	1,8	0,5	0	0,0	0,0	Prostate gland
C62	89	0,6	0,2	0	0,0	0,0	Testis
C63	48	0,3	0,1	0	0,0	0,0	Other and unspecified male genital organs
C64	182	1,3	0,4	113	0,7	0,2	Kidney
C65	2	0	0	2	0,0	0,0	Renal Pelvis
C66	2	0	0	0	0,0	0,0	Ureter
C67	657	4,8	1,3	227	1,4	0,4	Bladder
C68	12	0,1	0	10	0,1	0,0	Other unspecified urinary organs
C69	93	0,7	0,2	100	0,6	0,2	Eye and adexa
C70	24	0,2	0	39	0,2	0,1	Meninges
C71	390	2,8	0,8	283	1,8	0,5	Brain
C72	21	0,2	0	15	0,1	0,0	Spinal cord and other parts of CNS
C73	154	1,1	0,3	487	3,0	0,9	Thyroid
C74	6	0	0	5	0,0	0,0	Adrenal gland
C75	13	0,1	0	10	0,1	0,0	Other endocrine and related
C76	376	2,7	0,7	296	1,8	0,5	Other and ill-defined sites
C77	61	0,4	0,1	38	0,2	0,1	Lymph nodes
C80	227	1,6	0,4	189	1,2	0,3	Unknown Primary site
C81	187	1,4	0,4	81	0,5	0,1	Hodgkin's disease
C83	9	0,1	0	9	0,1	0,0	Diffuse non hodgkin lymphoma
C85	470	3,4	0,9	276	1,7	0,5	Non hodgkin lymphoma
C90	98	0,7	0,2	76	0,5	0,1	Multiple myeloma
C91	323	2,3	0,6	172	1,1	0,3	Leukemia/lymphoid
C92	350	2,5	0,7	237	1,5	0,4	Leukemia/myeloid
C94	0	0	0	0	0,0	0,0	Other leukemia
C95	94	0,7	0,2	61	0,4	0,1	Leukemia unspecified
TOTAL	13789	100	26,5	16013	100,0	29,2	
